# Educating healthcare professionals on interprofessional counseling in integrative oncology: development and evaluation protocol of the blended-learning program INSIGHT

**DOI:** 10.3389/fmed.2026.1823754

**Published:** 2026-06-30

**Authors:** Dominik Dupont, Andreas Schmitt, Regina Stolz, Nicole Folger, Jan Valentini, Stefanie Joos, Cornelia Mahler

**Affiliations:** 1 Department of Nursing Science, Institute of Health Sciences, University Hospital and Faculty of Medicine Tuebingen, Tuebingen, Germany; 2 Institute of General Practice and Interprofessional Care, University Hospital and Faculty of Medicine Tuebingen, Tuebingen, Germany; 3 Tübingen Center for Continuing Education, University of Tuebingen, Tuebingen, Germany

**Keywords:** adult oncology, complementary and integrative healthcare, complementary medicine, integrative nursing, interprofessional counseling, interprofessional education, oncology

## Abstract

**Introduction:**

Complementary and integrative healthcare (CIH) is a growing field of healthcare services in the German general population. About 40–80% of oncological patients use or would like to receive integrative supportive cancer care, depending on the tumor entity. However, health professionals often report that they are not adequately trained to provide evidence-based information on potential interventions. The objective of this study was to develop and evaluate a blended learning educational program for healthcare providers regarding integrative oncology, interprofessional counseling, and communication competencies on a healthcare provider level.

**Methods and analysis:**

After the investigation of preliminary studies, the curriculum of the educational program “Interprofessional Evidence-Based Education Program for Integrative Health Care in Oncological Counseling” (INSIGHT) was developed in an iterative process by nurses, physicians and researchers with expertise in the field of integrative oncology. A prospective, non-controlled, observational pre-post evaluation design using several validated questionnaires on sociodemographics and multiple dimensions of interprofessionality (UWE-IP, ISVS9a/b, EPIS-G) will be conducted.

**Discussion:**

The INSIGHT curriculum addresses insufficient clinician training in integrative oncology counseling despite high patient demand. By combining evidence-based CIH-education with interprofessional blended learning, it aims to improve knowledge, collaboration, and patient-centered care. If positively evaluated, the INSIGHT curriculum could serve as a scalable model for integrating evidence-based CIH education into oncology training programs and cancer center structures.

## 1 Introduction

Complementary and integrative healthcare (CIH) is a growing field of healthcare services in the German general population. A systematic review as well as a study investigating international survey data stated that 40–80% of cancer patients, depending on the tumor entity, would like to receive accompanying CIH (1, 2). The German national guideline on complementary medicine in oncology emphasizes the increasing evidence for various integrative interventions. The guideline advocates to take cancer patient’s history regarding the application of complementary measures followed by a consensus-based recommendation to counsel patients on its use as well as on potential interactions with medication (3). In addition, the guideline highlights the relevance of ensuring that multiprofessional teams are adequately qualified to provide state-of-the-art integrative symptom management. Nevertheless, current practice points to a lack of evidence-based structured educational programs for health-care professionals on counseling on CIH (3). An online survey study conducted with cancer care providers of multiple professions underlined the necessity of flexible educational options including scientific evidence as well as case analysis (4). Recent studies investigated the training needs of postgraduate general practitioners and general practice postgraduate trainees. The results indicated that integrating competencies of CIH is highly relevant, and the regular integration of CIH in a postgraduate family medicine program should be considered (5–7). A review indicates that nurses generally hold positive attitudes toward CIH. However, many report feeling insufficiently comfortable discussing CIH with their patients (8). In a survey study that examined healthcare professionals’ attitudes and educational disparities in German university hospitals, the results highlighted that these professionals subjectively lack sufficient training to counsel patients on CIH. They emphasized that targeted educational interventions are imperative to address the unmet needs of patients and professionals alike (9). A recent survey of outpatient care services in Germany also revealed both a high level of interest and a need for systematic continuing education (10).

In addition, many symptoms that cancer patients face, such as skin reactions (11), mucositis (12), chemotherapy-induced peripheral neuropathy (13), pain (11, 14), sleep disorders (15), and fatigue (11, 14), may be positively influenced by (naturopathic) nursing interventions (16). Although to date rarely evidence-based, these nursing interventions are often applied by experienced oncological nurses for symptom management. However, physicians are often not aware of the potential these nursing interventions have. This highlights the need for evidence-based interprofessional training and educational programs in CIH to address patients’ needs.

Educational programs that take an evidence-based approach to addressing complementary medicine and naturopathic nursing interventions for oncological patients in an interprofessional setting are rare in German-speaking countries (17). They predominantly address lifestyle-related health interventions and complementary medicine but lack the inclusion of nursing interventions. None of the existing programs explicitly teach how to collaborate in an interprofessional counselling setting within the CIH field. Most continuing education programs in the CIH field are tailored to a single profession, either nurses or physicians.

For the nursing profession, postgraduate qualification programs focus on integrative and complementary interventions that in particular address the management of specific symptoms, e.g., non-pharmaceutical pain treatment. Furthermore, there are educational programs which specifically focus on various integrative nursing interventions, such as aromatherapy, topical nursing interventions or anthroposophical nursing. All these educational programs prioritize one or several interventions, have heterogeneous admission regulations, and the duration and qualification levels do not adhere to standardized quality assurance with an evidence-based set of methods. However, none of these courses focus on an evidence-based, academic approach or result in a university certificate. A literature review investigating the relevance of CIH in courses targeting nursing students in Europe underlined the relevance of including communicational aspects and hands-on clinical skills in educational programs (18). After a pilot study exploring the integration of CIH in nursing education in Europe (19) a group of researchers from Denmark, Iceland, Sweden and the Netherlands developed a handbook of integrative nursing targeting educational staff in nursing professions (20).

A comparable picture emerges within medical postgraduate programs. In 2013, only 23% of physicians surveyed at a university hospital in Germany reported being satisfied with their level of information on complementary and alternative medicine (21). Only few accredited educational programs are recognized by the German federal medical councils, such as training in naturopathy, acupuncture, manual medicine, physical therapy, balneology and medical climatotherapy, nutritional medicine and sports medicine (8). These programs do not specifically focus on cancer patients and do not include interprofessional aspects. With the exception of naturopathy and acupuncture, there is also a lack of emphasis on CIH approaches. Furthermore, there are intervention-based training courses targeting physicians with multiple medical specializations. Additionally, intervention-based training programs are available for physicians from various medical specialties, primarily organized by professional societies such as the German Society of Traditional Chinese Medicine and the German Society of Anthroposophical Physicians. No other explicit interprofessional programs focused on counseling on integrative oncological medical and nursing procedures, culminating in a university-level certificate, were identified in German-speaking countries.

Individually tailored counseling which addresses biopsychosocial needs of oncological patients proved to be essential to educate patients about integrative medical and nursing interventions (22). Therefore, there is a significant need for educational programs which develop professional competence within a reasonable timeframe and incorporate both integrative medicine and nursing, as well as promote interprofessional collaboration and counseling in accordance with the guideline set forth at the national level (3).

Initial studies, such as the KOKON (Kompetenznetz für Komplementärmedizin in der Onkologie) consultation training intervention (18) and the CONGO (Complementary Nursing in Gynecologic Oncology) project (2014–2017) (16) began to shape educational programs on CIH in Germany. The multicenter CCC-Integrativ controlled implementation study which followed (2019–2023) demonstrated that a structured and interprofessional counseling service on CIH at four Comprehensive Cancer Centers (CCCs) in Germany targeting cancer patients had positive effects on patient activation, measured by PAM-13 as primary outcome. Participating CCCs were embedded in the University Hospital health services in Tuebingen, Freiburg, Heidelberg and Ulm, Germany (23). In order to maintain the counselling service established in CCC-Integrativ and to disseminate the service in additional CCCs the existing training program needed to be adapted to ensure and sustain evidence-based counselling.

The objective of the present study is to develop and evaluate the INSIGHT (Interprofessional Evidence-Based Education Program for Integrative Health Care in Oncological Counseling) blended learning educational program, focusing on integrative oncology, interprofessional counseling, and communication competencies at the healthcare provider level. INSIGHT advances the educational reasoning of the CCC-Integrativ project and is designed to empower healthcare professionals to counsel oncological patients on CIH. This program represents the first interprofessional initiative in integrative oncology counseling that leads to a university-level certificate.

## 2 Methods and analysis

### 2.1 Development of the educational program INSIGHT

For the development of the training program in the CCC-Integrativ study, curricula from previously conducted training programs were reviewed for relevant content: The educational intervention KOKON which includes a handbook with examples from real life consultations, e-learning modules with reviews, videos and CIH summaries in German as well as on-site skills trainings, was systematically analyzed (24). In addition, findings from the CONGO project, which focused on nursing interventions in supportive cancer care (16), a clinician-led consultation service (25) and current literature on the educational needs of cancer care providers (4) were reviewed. This resulted in the CCC-Integrativ educational program and was designed to train interprofessional teams (physician and nurse). This educational program covered eight core topics: Introduction into CIH and its evidence, communication skills, interprofessional skills, lifestyle measures as, e.g., nutrition, exercise and stress management, phytotherapy, naturopathic nursing interventions, acupressure and case studies (26). Building on the CCC-Integrativ educational program, INSIGHT was subsequently developed using a modified Kern curriculum approach (27). INSIGHT represents an advancement of the original program by incorporating findings from the CCC-Integrativ main study (23) and adapting the curriculum for structured academic continuing education.

A blended learning approach was selected to guarantee a substantial degree of flexibility for participants engaged in full-time employment or private obligations, while ensuring accessibility for individuals residing at a considerable distance from the University of Tübingen. Blended-learning approaches were chosen as they are associated with an increased short-term student-satisfaction and may improve critical thinking among students in healthcare professions (28). A workload of 300 h in total was deemed appropriate, in order to fulfill learning objectives of the program. Learning objectives were developed in alignment with the German national competence-based learning objectives catalog for medicine (NKLM) (29) and the German qualification framework (30). They are organized in two principal categories: Professional competence, encompassing theoretical and practical capabilities on CIH and interpersonal competence, referring to counseling and communication skills. For each module, specific learning objectives were defined, explicitly differentiating between the development of professional and interpersonal competencies.

The content of the INSIGHT-program was meticulously selected by an interprofessional panel of experts to guarantee its comprehensive coverage of the essential aspects of CIH within oncology. This iterative process was carried out by a visceral surgeon with a special focus in integrative oncology, a nursing scientist with a special focus in integrative topical applications and a health services researcher with a focus on palliative care nursing. All healthcare professionals had several years of experience in patient care and/or research. The interprofessional panel clustered topics and developed a schedule allocating the topics into a coherent and logical order over the time span of the course. Furthermore, there were regular reviews of the development process, the planned content of INSIGHT and the mode of delivery in the blended-learning program (on-site lectures or training sessions, webinars, asynchronous e-learning content) with experts in CIH research and practice. The expert group consisted of a senior professor of nursing science, a senior professor of general practice, a senior professor of visceral surgery with an additional qualification in drug-based tumor therapy and a senior physician with a focus on general practice and Traditional Chinese Medicine.

The content and development phases of INSIGHT were presented on an expert meeting of the “Forum for University Working Groups on Naturopathy and Integrative Medicine” in Germany, which yielded additional insights to a well-tailored educational program. After the final determination of the thematic fields of INSIGHT, further consultations were conducted with experts and potential lecturers for the program in the respective topics of CIH and medical disciplines.

INSIGHT as an educational program is incorporated in the continuing education department of the University of Tuebingen. The structural integration of the training program into the continuing education department of the University of Tübingen, a permanent institutional framework for academic continuing education at the university, enables the awarding of a university certificate and ensures the sustainable implementation of the program independent of project-based funding. After completing an examination, graduates of INSIGHT receive a Certificate of Advanced Studies comprising 10 European Credit Transfer and Accumulation System (ECTS) points. The target group of INSIGHT is comprised of physicians, nurses, and therapeutic professionals working in oncology settings or related fields of healthcare.

To successfully complete the CAS as a scientific educational program, participants are required to submit a written assignment based on an oncological case. The assignment comprises a structured case analysis, identification of counselling priorities, and a differentiated description of the counselling approach. Building on the content of the CAS, appropriate integrative interventions are discussed, and supplemented by a focused appraisal for one selected, less familiar intervention. This appraisal includes a brief literature search, a synthesis of key findings, their implications for counselling, and a reflective evaluation of the implemented interventions.

#### 2.1.1 Objectives of the educational program

The educational program is designed to enhance participants competence to deliver interprofessional counseling on evidence-based integrative and complementary interventions in oncological health care within their respective clinical contexts. This refers to the successful completion of a structured, university-based continuing education program that strengthens evidence-based CIH knowledge, interprofessional communication and collaboration skills, and the ability to systematically plan and reflect on counseling processes. Participants are trained to conduct consultations using a targeted and patient-centered approach, to utilize appropriate informational materials, and to continuously evaluate counseling objectives. The curriculum equips graduates with the competencies needed to establish and provide an interprofessional integrative consultation service within their institutions and is designed for direct transfer into professional practice.

#### 2.1.2 Structure of the program

**Figure 1:**
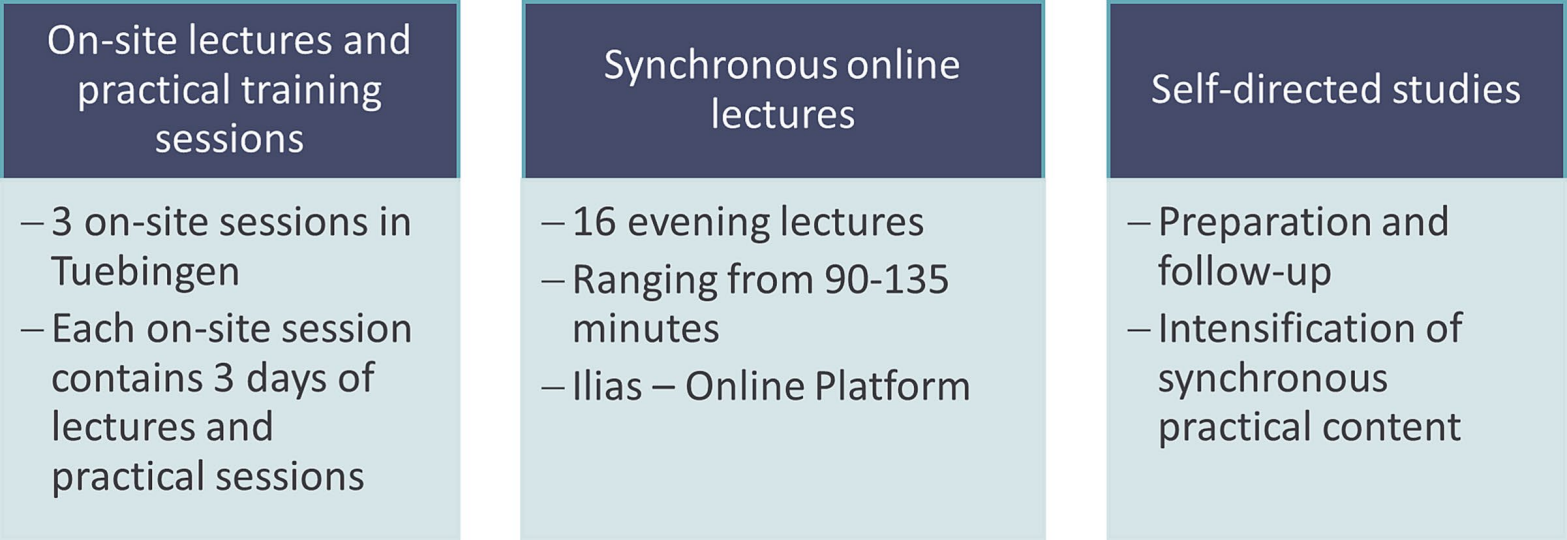
Structure of the educational program.

The program was developed as a blended learning format and comprises of three interrelated and alternating components that constitute the overall workload: on-site lectures combined with practical training sessions, synchronous online lectures and self-directed individual studies incorporating e-learning elements (see Figure 1).

Synchronous online lectures are delivered via the Zoom videoconferencing platform. Course content is grounded in the current state of evidence on CIH [e.g., national guideline (3)], as well as findings from previously published studies in this field (16, 23, 24).

The e-learning components of the educational program are delivered via the ILIAS online learning platform of the University of Tuebingen. The asynchronous e-learning platform will be structured chronologically and follows a coherent, logically sequenced progression of content. It comprises recommended literature, lecture slides, supplementary materials and video-based content addressing common oncological symptoms and integrative interventions of supportive cancer care. The online phases alternate with the on-site phases. In the online phases that precede each on-site phase, theoretical content relevant for the upcoming in-person sessions is delivered.

The educational content of the program, delivered in on-site, online and asynchronous modalities, focuses on interventions relevant to counseling, with particular emphasis on symptom management in oncological care. Core symptom domains include chemotherapy-induced peripheral neuropathy, palmar-plantar erythrodysesthesia/hand-foot syndrome, dermatologic toxicity, lymphedema, xerostomia, mucositis, pruritus, anxiety, fatigue, hot flashes, irritative cough, insomnia, pain, diarrhea, dysgeusia, nausea and vomiting, constipation, singultus, cancer-associated anorexia/cachexia, cystitis.

#### 2.1.3 On-site lectures and practical training sessions

The on-site lectures provide a general introduction to INSIGHT, principles of interprofessional collaboration, and foundational content on phytotherapy including pharmacological interactions between phytotherapeutic agents and oncological therapies. Additional topics include relaxation techniques and their evidence-base, as well as nutrition, malnutrition and cancer-related cachexia.

The practical training sessions comprise guided meditation, yoga and mindfulness-based-stress-reduction (MBSR) as along with hands-on sessions focusing on topical nursing interventions, case-based learning, and acupressure with emphasis on the symptoms addressed in the curriculum.

Particular attention is given to practice-oriented case discussions and the processes underlying interprofessional counselling. These case discussions are based on oncology-specific case vignettes and focus on the concrete realization of interprofessional counselling in everyday practice. All participating professions are expected to be familiar with the full range of course topics. Within the case discussions and counselling exercises, profession-specific emphases for nurses and physicians naturally emerge, informed by the preceding content-oriented learning units and the respective professional cultures. The interprofessional dynamics, including role clarification and collaboration processes, are explicitly facilitated and guided by an interprofessional teaching team consisting of a physician and a nurse.

#### 2.1.4 Synchronous online lectures

The synchronous online lectures, delivered via the Zoom videoconferencing platform, include an initial launch event comprising an introduction to integrative medicine and integrative nursing as well as an overview of the German national guidelines and available information resources on integrative cancer care. Additional evening lectures will address the following thematic areas:

1. Communication: motivational interviewing, core elements of patient counseling, intercultural care
2. Phytotherapy: cannabis, mistletoe, various other phytotherapeutics; oncological therapies and their potential interaction profiles; secondary phytochemicals and pharmacological interactions; CIH in radiation therapy and anthroposophical medicine
3. Naturopathic nursing interventions: Kneipp interventions and aromatherapy
4. Lifestyle interventions: therapeutic exercise, nutrition, fasting, nutritional supplements and circadian rhythm
5. Traditional Chinese Medicine: introduction and current evidence base of Traditional Chinese Medicine

#### 2.1.5 Self-directed studies

The self-directed study component, delivered via the e-learning platform, comprises contextual background materials, lecture slides, recommended literature, and asynchronous video content demonstrating acupressure points and evidence-based symptom management guidelines addressing the aforementioned symptoms.

### 2.2 Evaluation protocol of the educational program INSIGHT

The development phase of the INSIGHT blended learning program was completed in May 2025, and the program was subsequently implemented at the Tübingen Center for Continuing Education of the University of Tuebingen, with the first cohort commencing in October 2025.

No pilot study was conducted. The preliminary study CCC-Integrativ (23), which incorporated an educational component and process evaluations, functioned as a pilot for the educational program INSIGHT. This internal data was used to advance the topics and contents, the didactic approaches, and the modes of delivery of the included contents (e.g., clarity of tasks and navigation in ILIAS).

#### 2.2.1 Recruitment and sample size

Participants for the evaluation study will be recruited from three consecutive INSIGHT cohorts (2025/2026, 2026/2027, and 2027/2028) Participation in the educational program is independent of participation in the evaluation study. As enrollment in INSIGHT is limited to 25 participants per cohort a maximum sample size of 75 participants can be achieved and no sample size calculation was conducted. Inclusion criteria for the evaluation study include a minimum age of 18 years, fluent command of the German language, and enrollment in INSIGHT. The study’s objectives and relevant information will be provided during the initial lecture of the program, which will take place during the online launch event. During this event, informed consent will be obtained and T0 data collection conducted.

Admission criteria for INSIGHT comprise completion of an academic degree in a health profession or a completed vocational training in a health profession with a minimum of 2 years of professional experience. In addition, a professional connection to oncology is required.

#### 2.2.2 Study design and data collection

The evaluation study is designed as a longitudinal, prospective, single-arm, non-controlled, non-randomized observational pre–post study with follow-up. Given the limited sample size resulting from the restricted enrollment of the educational program, an exploratory analytical approach is adopted. Data will be collected at three time points per cohort: baseline (T0) on the introductory day of the program, at program completion (T1), and at a 6-month follow-up after completion (T2).

At T0, during the first event of the educational program, sociodemographic data will be collected using a standardized questionnaire to characterize the study population. Collected variables include age (categorized), gender, and stratified professional experience. Healthcare profession and professional role within the workplace (e.g., junior or senior physician) will also be assessed. Data collection will be conducted using the online platform SoSci Survey (version 3.8.00) and during the individual courses as well as T0, T1, and T2 a specific timeframe is reserved for the participation in the questionnaires. To support complete data entry, reminder prompts for unanswered items are integrated into the online questionnaire platform.

As an interprofessional educational program focusing on interprofessional counseling in integrative oncology, the primary objective of the evaluation is to examine interprofessional parameters as well as changes in self-perception on professional knowledge and competence on CIH, as detailed below. Given that the curriculum is interprofessional in both its mode of delivery and its intended application, questionnaires addressing interprofessional dimensions constitute the fundamental component of the evaluation approach.

##### 2.2.2.1 Individual course evaluation

Individual courses within the educational program are evaluated using a brief participant satisfaction survey. This survey comprises six items addressing various dimensions of the courses, including its informational content, practical relevance, motivational impact, perceived level of difficulty, effect on participants’ competence, and organizational quality. Respondents indicate their level of agreement with each statement on a five-point Likert scale ranging from “strongly agree” to “strongly disagree.” In addition to these closed-ended items, participants are invited to elaborate on their ratings and to provide suggestions or requests for future courses through open-ended questions. This approach allows for both quantitative and qualitative insights into participants’ perceptions and experiences. This formative evaluation enables timely responses to participants’ feedback and supports iterative conceptual refinements for subsequent cohorts. Participation in this component of the evaluation is determined on a course-by-course basis.

##### 2.2.2.2 Self-perception on professional knowledge and competence on CIH

To contextualize the competence improvement assessment following each individual course, at T0, T1 and T2 the self-perception of professional knowledge and competence in CIH will be investigated using a single-item measure. Participants will be asked, “How would you rate your current professional knowledge and competence in integrative oncology?” Responses were indicated on a slider scale ranging from 0 (“very low”) to 100 (“very high”).

##### 2.2.2.3 German version of the University of the West of England Interprofessional Questionnaire

This questionnaire was developed for the longitudinal evaluation of interprofessional curricula and comprises four scales assessing key interprofessional dimensions which will all be assessed at T0, T1 and T2. The Communication and Teamwork scale captures participants’ experiences with communication and teamwork in group settings. This scale does not include a neutral response option, based on the assumption that all respondents have prior experience in this domain. The Interprofessional Learning scale assesses participants’ attitudes toward interprofessional learning as well as perceptions of the timing of educational interventions. The Interprofessional Interaction scale addresses perceptions of the quality of interprofessional interactions in healthcare work environments. Both the Interprofessional Learning and Interprofessional Interaction scales consist of nine items each. The fourth scale, Interprofessional Relations, comprises eight items and evaluates relational aspects of interprofessional collaboration. With the exception of the Communication and Teamwork scale, all items are rated on a five-point Likert scale ranging from 1 (strongly agree) to 5 (strongly disagree). Scale scores reflect participants’ attitudes toward the assessed domains and differentiate between positive, neutral, and negative orientations (31). The validity, reliability, and psychometric properties of the German translation were examined in a study conducted in 2016, demonstrating good psychometric quality, with internal consistency coefficients ranging from 0.75 to 0.90 and a well-fitting underlying factor structure (32). The UWE-IP was selected because it captures key dimensions of interprofessional learning and collaboration that are directly addressed in the counseling and communication components of the INSIGHT learning objectives (see Supplementary File 1).

##### 2.2.2.4 German version of the interprofessional socialization and valuing scale 9a/b

This questionnaire focuses on socialization processes within the work environment, with particular emphasis on healthcare teams and will be collected during T0, T1 and T2. Following the initial publication of a 21-item version, a psychometrically equivalent 9-item version with pre- and post-test specifications was validated in English (33). The German version of the original 21-item scale was validated in 2022 and demonstrated high internal consistency (Cronbach’s *α* = 0.90) (34). To contribute to the ongoing validation efforts at the University Hospitals of Heidelberg and Tübingen, the ISVS-9a/b will be applied in the present study. The ISVS-9a/b was chosen because it reflects the program’s aim of fostering interprofessional socialization in the context of integrative oncology counseling and directly corresponds to the interprofessional collaboration learning objectives.

##### 2.2.2.5 German version of the extended professional identity scale

This scale operationalizes Extended Professional Identity Theory, which builds on concepts derived from social identity theory and identity theory. It comprises three subscales assessing interprofessional belonging, interprofessional commitment, and interprofessional beliefs (35). The German version demonstrates good internal consistency (Cronbach’s *α* = 0.89) and consists of 12 items distributed across the three aforementioned subscales (36). The EPIS-G will be collected at T0, T1, and T2. The EPIS-G was included to capture changes in professional identity (belonging, commitment and beliefs), which may be influenced by participants repeated engagement in interprofessional learning activities, including longitudinal work on case examples and exposure to perspectives from different healthcare professions. In line with these learning activities, we aim to explore whether participation in the program is associated with changes in participants professional identity with regard to interprofessional collaboration.

#### 2.2.3 Data analysis

Sociodemographic variables, individual course evaluation and self-perception on professional knowledge and competence on CIH will be analyzed descriptively. Scores obtained from the applied instruments (UWE-IP, ISVS 9A/9B, and EPIS-G) will be compared across the three measurement time points within the same sample. Primary analyses will be descriptive and will include measures of central tendency (means, medians), measures of dispersion, and graphical visualization of temporal trends.

The distributional properties of the data will be examined using the Shapiro–Wilk test in conjunction with visual inspection of Q–Q plots. Exploratory within-subject comparisons across time points (T0, T1, and T2) will be conducted to assess potential changes in scores over time. For normally distributed data, repeated-measures analysis of variance (ANOVA) will be applied, whereas the Friedman test will be used for non-normally distributed data. Given the limited sample size, all inferential analyses will be interpreted in an exploratory manner. Where notable differences are observed, *post hoc* analyses (e.g., pairwise Wilcoxon signed-rank tests with Bonferroni correction) may be performed to further describe changes between specific time points.

Attrition between T0, T1, and follow-up (T2) will be reported using flow diagrams and descriptive statistics (e.g., number and proportion of participants at each time point, reasons for drop-out where available). For within-subject analyses of change over time, only participants with data at the relevant time points will be included. Comparisons of key baseline characteristics between completers and non-completers will be considered to explore potential attrition bias. Missing data will be handled using a complete case approach for analyses of within-subject change over time. No imputation across time points or at the participant level is planned, given the exploratory design and limited sample size. At the item level, person-mean imputation within a scale will be considered if missing responses do not exceed a predefined threshold and the extent and patterns of missing data will be reported descriptively.

## 3 Ethical considerations and dissemination

Prior to participation, study-specific information regarding study procedures, data protection, and the right to withdraw will be provided, and written informed consent will be obtained from all participants. Participation is voluntary, and participants may withdraw from the study at any time without providing a reason. Pseudonymized data may be deleted upon request by referencing the individual pseudonym.

Data collection will be conducted using the online platform SoSci Survey (version 3.8.00). All data will be stored on secure servers of the University Hospital of Tübingen. The study will be conducted in accordance with the Declaration of Helsinki as well as applicable European and German national regulations. Ethical approval was obtained from the Ethics Committee of the Medical Faculty of the University of Tübingen (Approval No. 2025-0617-BO).

In addition to feedback provided to the study population, anonymized study results will be disseminated through peer-reviewed scientific journals and presentations at scientific conferences.

## 4 Discussion

The development of the INSIGHT curriculum responds to a well-documented gap between the high prevalence of CIH use among oncology patients and the limited training of healthcare professionals in evidence-based counseling targeting integrative oncology (9). International data indicate that approximately 40–80% of oncological patients use or consider CIH interventions, depending on tumor entity and study design (2). Despite this widespread use, many clinicians in Germany as well as in other countries report insufficient knowledge and low confidence in advising patients on the potential benefits, risks, and interactions of integrative approaches (9, 37). Professional organizations and guidelines such as the American Society of Clinical Oncology or the German national guideline on complementary medicine in oncology emphasize the importance of structured education and interdisciplinary collaboration to ensure safe, patient-centered integrative oncology care (3, 38).

The INSIGHT program contributes to this field by integrating evidence-based CIH knowledge with interprofessional communication training in a blended-learning format. Interprofessional education may be a key strategy to improve collaboration and quality of care in complex clinical settings such as oncology (39, 40). Similarly, blended learning has shown effectiveness in continuing medical education by improving learner engagement, the autonomy of learners, satisfaction and knowledge retention compared with traditional teaching formats (41, 42). Combining these approaches may therefore be particularly suitable for integrative oncology counseling, which requires both critical appraisal of heterogeneous evidence, evaluating the inclusion of consensus-based interventions with lacking evidence in counseling and collaborative decision-making across professional boundaries. The World Health Organization’s Framework for Action on Interprofessional Education and Collaborative Practice positions interprofessional education as a key strategy to build a “collaborative practice-ready” health workforce and strengthen health systems through effective teamwork across various health professions (43). The INSIGHT program contributes to this goal by operationalizing this framework, integrating evidence-based CIH content with interprofessional communication training in a blended-learning format.

The planned prospective pre-post evaluation using validated interprofessional instruments allows an initial assessment of changes in self-perceived competencies and attitudes. However, the non-controlled design limits causal inference, and reliance on self-report measures may introduce social desirability bias or response-shift effects. Long-term follow-up is also needed to determine whether educational gains translate into sustained practice change and improved interprofessional collaboration. Performance-based assessments or observational examinations, such as objective structured clinical examinations, may offer a more objective evaluation option increasing the validity of the findings. However, after intensive discussion within the research team, the decision was made not to implement such assessments due to the substantial resources required for their administration and execution. Participants travel from across Germany, so on-site time is deliberately kept to a minimum to support compatibility with work and everyday life, with a clear focus on the acquisition of practical skills. Therefore, participant-reported assessments were chosen for the evaluation of the educational program. While the curriculum is explicitly designed for direct transfer into professional practice and to equip participants with competencies for interprofessional CIH counseling, the present evaluation focuses on self-reported competence and interprofessional outcomes and does not include performance-based assessments of counseling in real-world clinical encounters, which limits the extent to which actual practice change can be investigated.

If positively evaluated, the INSIGHT curriculum could serve as a scalable model for integrating evidence-based CIH education into oncology training programs and cancer center structures. Such initiatives may contribute to safer integrative care, improved communication about CIH use, and more informed shared decision-making, which are increasingly recognized as essential components of high-quality oncology care.
