# Supplementary material for: Educating healthcare professionals on interprofessional counseling in integrative oncology: development and evaluation protocol of the blended-learning program INSIGHT
Source: Front Med (Lausanne). 2026 Jun 30;13:1823754. doi: 10.3389/fmed.2026.1823754 (PMC13364837; doi:10.3389/fmed.2026.1823754)
Supplement: Supplementary file 2 [file Data_Sheet_1.PDF]

Supplementary File: Structured presentation of modules, learning objectives, instructional methods, and duration of the INSIGHT blended-learning program

Thematic area	Included topics	Learning objectives	Instructional methods	Background of the lecturers and trainers	Workload
**Introduction**	– Terminology, evidence base, historical development, and key concepts (e.g. self-efficacy, interprofessional collaboration) in the context of integrative healthcare<br>– Overview of symptoms and typical counselling needs in oncological care<br>– Presentation of the structure of the educational program	**Professional competencies**<br>By the end of the course, participants will be able to:<br>1. describe key terms, concepts and the development of integrative oncology;<br>2. explain and contextualise relevant theoretical concepts;<br>3. independently search for and critically appraise relevant specialist information.<br><br>**Counseling and communication competencies**<br>By the end of the course, participants will be able to:<br>1. reflect on and share their own prior experiences and expectations;<br>2. participate actively and appreciatively in group discussions;<br>3. contribute constructively to an interprofessional learning environment.	**On-site and online:** lectures, seminars<br><br>**Self-directed study time supported by:** Content-related texts	Professor of Nursing Science, Palliative Care Nurse, senior visceral Surgeon, Nursing Scientist, Oncologist	16
**Interprofessional collaboration**	– Fundamental concepts and principles underlying interprofessional collaboration,<br>– Teambuilding<br>– ComSkill and Team C	**Professional competencies**<br>By the end of the course, participants will be able to:<br>1. describe fundamental concepts of interprofessional collaboration.<br>2. identify and apply principles and models of effective teamwork (e.g., Team C).<br>3. explain key elements of professional interprofessional communication (e.g., ComSkill).<br>**Counseling and communication competencies**<br>By the end of the course, participants will be able to:<br>1. conduct conversations in interprofessional contexts in a structured and audience-appropriate manner.<br>2. recognize and implement communication-enhancing behaviors within the team.<br>3. reflect on the specific features and challenges of interprofessional counseling.	**On-site and online:** seminars, webinars, case-based work, role plays, and practical exercises<br><br>**Self-directed study time supported by**: content-related texts, supplementary literature, webcasts	Nursing expert with a focus on interprofessional collaboration	12
**Communication**	– Core elements of patient counselling and structuring counselling interventions	**Professional competencies**<br>By the end of the course, participants will be able to:<br>1. identify and structure key elements and models of professional counselling processes;<br>2. explain the basics of motivational interviewing and apply them to counselling situations;	**On-site and online:** seminars, webinars, case-based work	Pulmonologist, Psycho-oncologist, Nurse with a training in pedagogics and international experience	12

	– Motivational interviewing, communicative challenges and conflict resolution<br>– Intercultural care	3. identify and contextualise communicative and intercultural challenges in counselling settings.<br>**Counselling and communication competencies**<br>By the end of the course, participants will be able to:<br>1. conduct counselling sessions in a structured, goal-oriented and resource-focused manner;<br>2. communicate in a de-escalating and solution-focused way in challenging conversations;<br>3. respond sensitively and reflectively to cultural differences in counselling contexts.	**Self-directed study time supported by**: content-related texts		
**Lifestyles: nutrition, physical activity, relaxation techniques**	**Physical activity**<br>– Evidence on physical activity in oncology<br>– Prehabilitation<br>– Physical activity in oncological diseases<br>**Nutrition**<br>– Principles of healthy nutrition, evidence in oncology, fasting, vitamins, dietary supplements, secondary plant compounds<br>– Nutrition in specific oncological diseases, malnutrition, cachexia<br>**Relaxation techniques**<br>– Introduction and definition of relaxation techniques<br>– Evidence base for relaxation techniques<br>– Meditation/mindfulness-based stress reduction, yoga, etc.<br>– Circadian Rhythm and regulative therapy	**Professional competencies**<br>By the end of the course, participants will be able to:<br>1. explain the evidence base on physical activity, nutrition and relaxation techniques in oncology;<br>2. describe prehabilitation approaches and exercise-based interventions in oncological diseases;<br>3. explain principles of healthy nutrition, including fasting, dietary supplements and secondary plant compounds, and relate them to oncological contexts;<br>4. formulate nutritional recommendations for specific oncological diseases and differentiate professionally between malnutrition and cachexia;<br>5. identify various relaxation techniques (e.g. MBSR, yoga), explain their effects and gain initial practical experience with them;<br>6. describe the concept of circadian rhythms and outline its relevance for oncological counselling.<br>**Counselling and communication competencies**<br>By the end of the course, participants will be able to:<br>1. integrate evidence-based information on physical activity, nutrition and relaxation into counselling sessions in a target group–appropriate manner;<br>2. motivate patients in a resource-oriented and empathetic way to adopt health-promoting lifestyle measures;<br>3. address uncertainties or misunderstandings related to nutrition and complementary methods in a professional manner.	**On-site and online:** lectures, seminars, case-based learning, practical exercises with self-experience<br>**Self-directed study time supported by**: Webcasts, content-related texts	sports scientist, senior physician specialized in internal medicine, physician specialized in nutritional medicine; nutrition scientist (ecotrophologist); professor of visceral surgery with an additional qualification in drug-based tumor therapy; professor of complementary medicine research with a background in psychology; visceral surgeon; yoga teacher	38

**Phytotherapy**	– Evidence base for herbal medicinal products in oncology, mechanisms of action, authorized medicinal products vs. dietary supplements<br>– Selected herbal medicinal products for specific symptoms, including mistletoe therapy and cannabis<br>– Medication safety and drug interactions – theory, key aspects, quality<br>– Basic principles of oncological therapies (chemotherapy, immunotherapy, endocrine therapy, interaction potential of selected drugs)<br>– Medication safety and drug interactions – practical exercises on interactions between dietary supplements/herbal medicinal products and antitumor therapies<br>– Specific features of anthroposophical medicine<br>– Integrative interventions in radiotherapy	**Professional competencies**<br>By the end of the course, participants will be able to:<br>1. explain the evidence base and mechanisms of action of herbal medicinal products and distinguish them from dietary supplements;<br>2. provide counselling on selected herbal medicinal products (including mistletoe therapy and cannabis) for oncological symptoms;<br>3. explain the basic principles and interaction potentials of oncological drugs (chemotherapy, immunotherapy, etc.);<br>4. assess drug interactions and risks in the context of herbal medicinal products and conventional therapies;<br>5. describe the specific features of anthroposophical medicine in oncological counselling.<br>**Counselling and communication competencies**<br>By the end of the course, participants will be able to:<br>1. counsel patients in a differentiated manner on herbal medicinal products, drug interactions and dietary supplements, taking into account benefits, risks and quality;<br>2. address uncertainties regarding the use of complementary and conventional medicines in a professional way;<br>3. communicate interprofessionally on medication-related issues and contribute responsibly to counselling	**On-site and online:** lectures, seminars, case-based learning and practical exercises using interaction databases<br>**Self-directed study time supported by:** webcasts, content-related texts	Professor of Haemato-oncology with additional qualification in naturopathy; Pharmacologist with specific training in drug interactions between antitumor therapies, phytotherapeutics and dietary supplements; Professor of Haematology and Oncology; Professor of Radiation Oncology; Visceral Surgeon	38
**Naturopathic nursing interventions**	– Introduction to the basics, evidence and mechanisms of action of	**Professional competencies**<br>By the end of the course, participants will be able to:<br>1. explain the basics, evidence and mechanisms of action of naturopathic nursing interventions;	**On-site and online:** lectures, seminars, case-based learning and	Kneipp specialist with a background in general practice, oncology nurse	40

	naturopathic nursing interventions<br>– Compresses and poultices<br>– Rubbing applications (f.e. oil rubs)<br>– Kneipp hydrotherapy methods<br>– Aromatherapy	2. describe compresses, poultices, oil rubs as well as aromatherapy as nursing interventions, explain their use in counselling practice and perform selected interventions;<br>3. explain Kneipp-based therapies and outline their possible applications in nursing.<br>**Counselling and communication competencies**<br>By the end of the course, participants will be able to:<br>1. competently counsel patients on naturopathic nursing interventions and clearly communicate their benefits and risks;<br>2. explain the practical application of compresses, poultices, oil rubs and aromatherapy in a clear and comprehensible way;<br>3. translate individual recommendations for Kneipp therapies and other naturopathic procedures into counselling practice.	practical exercises with self-experience<br>**Self-directed study time supported by:** webcasts, content-related texts	specialists, nurse specialists in integrative oncology, anthroposophic nurse specialist, nurse aromatherapy expert	
**Acupressure**	– Introduction and evidence base of Traditional Chinese Medicine (TCM) and acupuncture<br>– Introduction to acupressure<br>– Symptom-oriented acupressure	**Professional competencies**<br>By the end of the course, participants will be able to:<br>1. explain the fundamentals and evidence base of Traditional Chinese Medicine (TCM) and acupuncture;<br>2. explain the principles of acupressure and outline its possible applications;<br>3. apply symptom-oriented acupressure methods and describe their effects in practice.<br>**Counselling and communication competencies**<br>By the end of the course, participants will be able to:<br>1. explain the basics and benefits of TCM and acupuncture to patients in an understandable way;<br>2. present acupressure as a therapeutic method in a clear and accessible manner and explain its application in counselling;<br>3. recommend individual acupressure techniques in a targeted way during counselling sessions.	**On-site and online:** lectures, seminars, case-based learning and practical exercises on the localisation and stimulation of acupressure points<br>**Self-directed study time supported by:** webcasts exercises on the localization of acupressure points, content-related texts	Senior physician with a focus on general practice and Traditional Chinese Medicine, senior physician with a focus on visceral surgery and acupuncture	24
**Counseling indications**	– Introduction to symptom-oriented counselling indications, specific counselling approaches, patient-individual selection and implementation of interventions, and	**Professional competencies**<br>By the end of the course, participants will be able to:<br>1. explain the structure and use of symptom-oriented counselling guides;<br>2. draft counselling letters and present the key content in a structured way;<br>3. explain the guidelines on complementary medicine in oncology as well as other relevant guidelines and outline their relevance for clinical practice;	**On-site and online:** seminars, case-based learning with case discussions, role plays and practical exercises on symptom clusters and counselling situations<br>**Self-directed study time supported by:** webcasts on relevant symptoms	Palliative care nurse with a background in health services research, senior visceral surgeon	70

	adaptation of counselling strategies<br>– Writing counselling letters<br>– Introduction to and in-depth work with the S3 guidelines on complementary medicine in oncology, supportive therapy in oncological patients and additional relevant guidelines<br>– Symptom-oriented counselling exercises using counselling guides	4. conduct symptom-oriented counselling sessions using the counselling guides.<br>**Counselling and communication competencies**<br>By the end of the course, participants will be able to:<br>1. write counselling letters in a clear, precise and target group–appropriate manner;<br>2. integrate counselling guides purposefully into conversations and adapt their use to individual needs;<br>3. conduct symptom-oriented counselling sessions and respond competently to patients' concerns.	and complementary interventions in oncology, information leaflets, topic guides, excerpts from clinical guidelines		

Information materials

Applied information leaflets on complementary interventions and scientific guidelines can be accessed via https://kompetenznetz-kokon.de/ (information leaflets) and https://www.leitlinienprogramm-onkologie.de/leitlinien/uebersicht (guidelines).

Examination

The final examination consists of a 15–20-page written assignment (workload 50 hours), in which participants analyze an oncological case, outline counselling priorities and processes, and discuss suitable integrative interventions based on the contents of the INSIGHT certificate program. In addition, they conduct focused evidence search on a new or only marginally covered intervention, critically appraise the findings, and reflect on the implemented or recommended measures, concluding with an overall assessment of outcomes and the program.
